# Effects of Gallic Acid on Endometrial Cancer Cells in Two and Three Dimensional Cell Culture Models

**DOI:** 10.31557/APJCP.2021.22.6.1745

**Published:** 2021-06

**Authors:** Muhammet Volkan Bulbul, Seda Karabulut, Mervenur Kalender, Ilknur Keskin

**Affiliations:** 1 *Department of Histology and Embryology, School of Medicine, Istanbul Medipol University, Istanbul, Turkey. *; 2 *Research Institute for Health Sciences and Technologies (SABITA), İstanbul, Medipol University, Istanbul, Turkey. *

**Keywords:** Spheroid, monolayer, MTT- caspase 3, apoptosis

## Abstract

**Background and Aim:**

Cell culture studies are an indispensable tools used to understand basic physiological, biophysical and biomolecular mechanisms. Although traditional two-dimensional (2D) cell culture models are more preferred in experimental studies, three-dimensional (3D) cell culture models, attract more attention due to several advantages including mimicking tumor physiology, biochemistry and biomechanics. We aimed to investigate the effects of Gallic Acid, an antimutagenic, antioxidant and anticarcinogenic agent, on both 2D and 3D cultured endometrial cancer cells for the first time.

**Methods:**

IC50 values were determined in 2D and 3D cultured endometrial cancer cells exposed to different doses of GA. In the 2D culture model exposed to GA, Caspase 3 expression levels were analyzed. In addition, the effect of GA on the migration of 2D cultured endometrium cancer cells was investigated.

**Results:**

IC50 value in the 3D model was found significantly higher than the 2D model. In 2D culture model, Caspase 3 expression and apoptosis was increased significantly in cells of GA exposed group compared to the control group. GA did not have a significant effect on the migration profile of cells.

**Conclusion:**

Gallic Acid is shown to induce apoptosis in Ishikawa cells via Caspase 3 activation. We demonstrated a significantly higher IC50 level in 3D model which provide evidence to prefer 3D models in drug-test trials. The data obtained in the current study will provide a basis for further experiments to analyze the effects of GA on endometrial cancer and to develop strategies for clinical treatment.

## Introduction

Endometrial cancer is one of the most common gynecological malignancies in the United States and is the fourth most common cancer in women after breast, lung, and colorectal cancer (Siegel et al., 2012) and the death rate has increased more than 100% in the past 20 years (Rose, 1996). Risk factors of endometrial cancer include estrogen therapy, early menarche, late menopause, infertility or ovulation failure, polycystic ovarian syndrome, advanced age, obesity, hypertension, diabetes mellitus and colorectal cancer without hereditary polyposis (Sorosky, 2008). Chemotherapy is an important part of treatment for many cancer types, and efforts to develop new anti-cancer drugs occupy one of the biggest areas in pharmaceutical industry (Chabner and Roberts, 2005). It is known that a wide variety of chemotherapeutic agents are used clinically to induce apoptosis in cancer cells, some of which are cisplatin, taxol and doxorubusin (Fuertes et al., 2003). In a study examining the effects of Cisplatin, Taxol and Doxorubusin individually and in combination on Ishikawa cells, these agents were shown to induce apoptosis in cancer cells by affecting PI3-K/Akt pathway, one of the important pathways regulating the cell cycle (Gagnon et al., 2008). However, it is a well known phenomenon that chemotherapeutics damage cancer cells, however, they also damage healthy cells, causing many side effects (Pearce et al., 2017). Gallic acid (3,4,5-trihydroxybenzoic acid; GA) is an organic phenolic compound found in natural plants and fruits such as sumac, grapes, green tea, oak bark, strawberries, lemons, bananas, pineapples, witch hazel and apple peels (Yeh and Yen, 2005). GA and its analogs, polyhydroxyphenolic compounds, have been reported to have many biological applications, including their usage as antioxidants, anti-mutagenic and anti-carcinogenic agents (You et al., 2010). Studies with several cancer cell lines have shown that GA induces apoptosis by inducing mitochondrial membrane loss, release of cytochrome c, and initiating caspase activation due to the formation of reactive oxygen species (Russell et al., 2012). GA’s anti-cancer activity has been reported in various cancer cells, including prostate, lung, stomach, colon, breast, cervical, and esophageal cancer(Faried et al., 2007). To date however, there has been no study investigating the anti-carcinogenic effect of GA on the Ishikawa cell line, which is an endometrial cancer cell line.

Almost all of the studies that investigate the cytotoxic effects of GA on cancer cells have traditionally been tested on two-dimensional (2D, monolayer) cell culture models in which the cells can not communicate with one another, except through lateral membrane connections (O’Brien et al., 2002). Although this in vitro cell model has provided valuable information about the mechanisms underlying malignant growth, it is not suitable to fully mimic in vivo tumors. It is well known that solid tumors grow on a three-dimensional (3D, spheroid) spatial plane, and cells in these tumors are subjected to non-homogeneous oxygen distributions and nutrients, as well as other physical and chemical stresses (Graziano and Preziosi, 2007). As a result, significant cellular heterogeneity may occur due to large micro-environmental variations in different parts of tumors. In areas where oxygen and nutrient sources are low, cell damage and even necrosis may develop (Nath and Devi, 2016). In the light of these data, it is clear that the growth of cancer cells on a 2D cell model, where all the cells are equally exposed to oxygen and nutrients, cannot be used to study all aspects of tumor biology. In addition, it is suggested that most of the differences in response to radio and chemotherapy observed between monolayer cells and those found in in vivo tumors may be the direct result of differences in spatial organization and cell to cell contacts(Santini and Rainaldi, 1999).

A study analysing the effects of Cisplatin, a cytotoxic agent, in 2D and 3D cell culture models of Hela cells, exposed at same doses and equal incubation times, showed that the inhibitor concentration was significantly increased from 7.5 micromolar in the 2D culture to 60 micromolar on 3D model. These results proved that multi-drug resistance was higher in 3D culture systems than 2D models, just like in vivo(Zeng et al., 2016).

As a result, studies with 3D in vitro models which are avascular tumor models have gained popularity(Cryan et al., 2019), and cytotoxic analyzes, cell to cell interactions, apoptotic-necrotic cells, etc. are investigated on these 3D avascular tumor models(Lin and Chang, 2008).

In the present study, we aimed to investigate the effect of GA on endometrial cancer cell lines, Ishikawa cells, on 2D and 3D culture models..

## Materials and Methods

Our experiments were carried out at the cell culture level. For this purpose, Ishikawa cells were cultured in 2D and 3D. MTT analysis was performed to reveal the cytotoxic effect of different dose concentrations (5, 10, 30, 50, 75, 100 μg / mL) of GA in both culture models at 24 hours. Then, the effect of IC_50_ value determined in the 2D model on the migration of Ishikawa cells was investigated. Finally, the relationship of the apoptotic effect of GA with the Caspase 3 pathway was revealed semi-quantitatively by the immunofluorescence staining method.

*Culture of Ishikawa Cells*

The human endometrium adenocarcinoma cell line (Ishikawa) was purchased from the European Authenticated Cell Cultures Collection (ECACC, 99040201).The cells were cultured in MEM (Thermo Fischer Scientific, 12492013), supplemented with 2mM Glutamine (Thermo Fischer Scientific, 25030081), 1% Non Essential Amino Acids (Gibco, 11140050), 1% penicillin-streptomycin (Sigma Aldrich, P4458) and 10% fetal bovine serum (Sigma Aldrich, F2442), and incubated at 37°C in a humidified atmosphere with 5% CO2. Cells were routinely sub-cultured using 0.25% trypsin/EDTA (Sigma Aldrich, T4049).

*Preparation of Gallic Acid*

The doses selected for the study were selected according to the reference of an experimental study investigating the effect of GA on MCF-7 cells, since there is no study evaluating the cytotoxic effect of GA on Ishikawa cells(Rezaei-Seresht et al., 2019). 

To prepare GA (Sigma Aldrich, CAS Number: 149-91-7) at different concentrations (5, 10, 30, 50, 75, 100 μg / mL), 20 mg of GA was dissolved in 1 ml of dimethyl sulfoxide (Sigma Aldrich, CAS Number: 67-68-5) and a highly concentrated stock solution was prepared. All doses were prepared by diluting the whole culture medium using fresh stock solution before the experiment.

*MTT cell viability Assay for 2D (Monolayer) Culture*

Ishikawa cells were seeded into each well of a 96-well microculture plate (Nest^®^) at a concentration of 3× 10^5 ^cells/well. The 96-well microculture plates were treated with various concentrations of GA (5, 10, 30, 50, 75, 100 μg / mL). Cells treated with % 0.1 TritonX-100 (Millipore, 108603) were used as the positive control group, and cells not treated with GA formed the negative control group. 

After 24 hours of incubation, medium- GA mixtures were aspirated. The wells were washed with PBS so as not to damage the cells. 200 microliters of MTT (Vybrant^® ^MTT Cell Proliferation Assay Kit) solution, diluted 1:20 in medium, was added to each well. MTT solutions were aspirated after 3 hours of incubation. Formazan salts were dissolved by adding 200 microliters of DMSO to each well. Measurements were made with a 570 nm SpectraMax i3 microplate reader device. The IC_50_ value was detected by a dose response inhibition panel graph at GraphPad Prism 6.0 software.

*MTT cell viability assay for 3D (Spheroids) Culture*

96-well plates were coated with 1% agarose in PBS. 10,000 cells were added to each well in 200 microliters of medium. Following the formation of spheroids 4 days later, spheroids were transferred to another 96-well plate via 200 micrometer pipette tips. Before and after the application of GA to each spheroid, their diameters were measured and normalized. MTT analysis was performed with the same protocol applied to 2D culture. Measurements were made with a 570 nm SpectraMax i3 microplate reader. The IC_50_ value was detected by a dose response inhibition panel graph at GraphPad Prism 6.0 software.

*Wound Healing and Migration Assay*

Culture-Insert 2 Well in µ-Dish 35 mm (ibidi, Cat.No 80206) was used for a wound healing and migration experiment. 3× 10^5^ cells were added in a 70 microliter medium each in two wells. These were incubated at 37°C and 5 % CO_2_ as usual. After appropriate cell attachment (24 hours) the Culture-Insert 2 Well was gently removed using sterile tweezers. IC_50_ GA was added to the experimental group cells in 2 ml. doses. The migration rate in both experimental and control group cells was displayed at 0, 4 and 24 hours. Wound closure rates were measured and recorded with the ImageJ software program.

*Caspase-3 expression*

Caspase-3 expressions were analysed by an immunofluorescence technique. Ishikawa cells were seeded into 8-well chamber (ibidi, Cat.No:80841) at a concentration of 3× 10^5^ cells/well. After cell attachment, IC_50_ GA was added to the experimental group cells in 200 microliters and incubated for 24 hours. After the incubation, for permeabilization and fixation, a 1: 1 ratio of methanol-acetone was added to the wells and held at -20 °C for 15 minutes. After permeabilization and fixation, washing was performed 3 times for 5 minutes each with pbs. Pbs containing 0.1% Tween20 (Sigma Aldrich, 9005-64-5), 5% normal goat serum (Sigma Aldrich, 566380) was used for blocking. After blocking for 2 hours at room temperature, washing was done twice for 5 minutes with pbs. The primary antibody Caspase-3 (Santa Cruz, sc-136219) was diluted 1: 100 in the antibody dilütion buffer (Sigma Aldrich, U3635), added to the wells, and incubated overnight at +4 °C. Then, washing was done 3 times for 5 minutes each with pbs. The secondary antibody Goat Anti-mouse Alexa Fluor 568 (abcam, ab175471), diluted 1: 200 in antibody dilution buffer was added and incubated for 3 hours at room temperature. Washing was done 3 times for 5 minutes each with pbs. The preparation was closed with Fluoromount with DAPI (Thermo Fischer Scientific, 00-4959-52). Images were recorded using the Zeiss LSM 800 confocal microscope. The ImageJ program was used for intensity measurements.

*Statistical Analysis*

All statistical analyses and graphics were performed using the GraphPad Prism 6.0 software program. The results of MTT analysis were evaluated with One way Anova and Tukey’s Multiple Comparisons statistics panels. Immunofluorescence staining results were evaluated with an Unpaired T test. Values below p <0.05 were considered significant for MTT analysis results in 2D and 3D cell culture. Values below p <0.05 were considered significant for the results of the intensity analysis determined in the immunofluorescence staining.

## Results

*Cytotoxic Activity in 2D (Monolayer) Culture*

**Figure 1:**
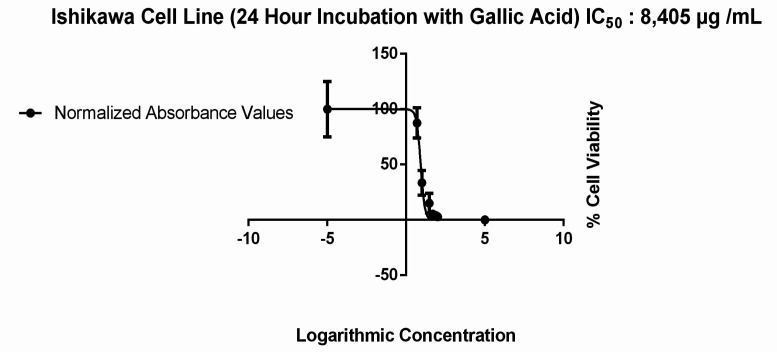
IC_50_ Value in 2D Cultured Ishikawa Cells Treated 24 hours with 6 Different (5, 10, 30, 50, 75, 100 μg / mL) Doses of GA

**Figure 2:**
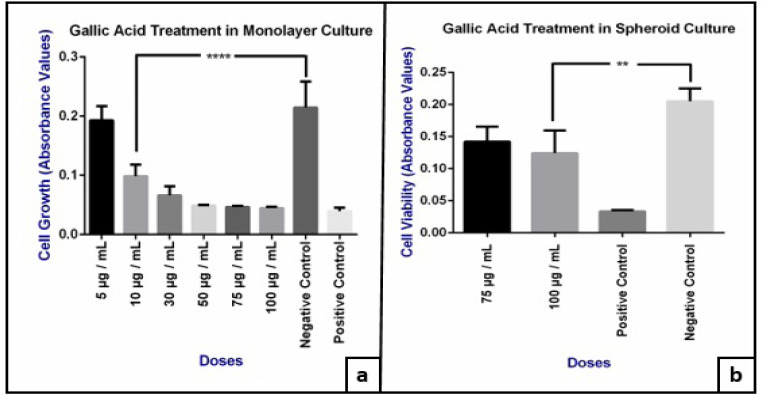
a, Effect of different doses of GA on the viability of 2D cultured Ishikawa cells (****: <0.0001); b, Effect of GA (75 and 100 μg/ml doses) on the viability of Ishikawa spheroids (**: < 0.005).

The viability of Ishikawa cells in 2D in vitro models that were treated with different doses of GA (5, 10, 30, 50, 75, 100 μg / mL) for 24 hours, was evaluated by MTT assay and IC_50_ values were calculated. We observed an IC_50_ value of 8.405 μg / mL (Figure 1) and a dose dependent decrease in cell viability in 2D culture model as shown in Figure 2-a (p= 0,0001). 

*Cytotoxic Activity in 3D (Spheroid) Culture*

**Figure 3:**
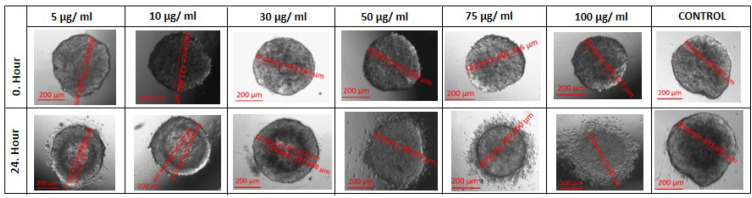
Difference in Spheroid Sizes after GA Exposure in Aall Experimental (5 to 100 μg/ ml) and Control Groups

**Figure 4:**
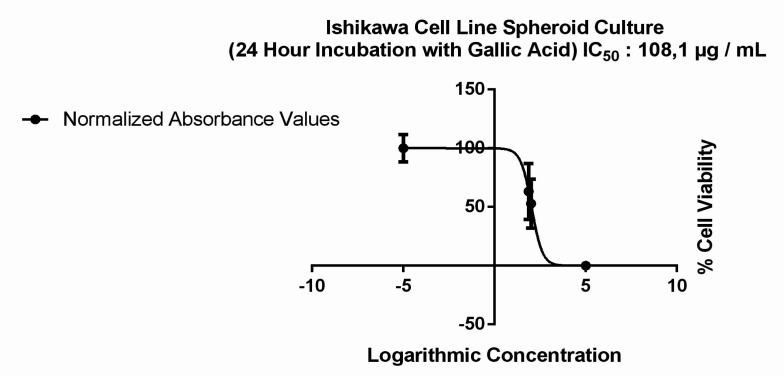
IC_50_ Value in Spheroid Cultured Ishikawa Cells Treated 24 hours with Two Different Doses (75 μg/ml and 100 μg/ml) of GA

MTT analysis was performed by applying the same doses to spheroids. GA had no effect on the size of spheroid forms between the doses of 5 to 50 μg/ml (Figure 3 and Table 1). However a significant dose dependent decrease were observed in cell viability only in 100 μg/ ml dose (p=0.006) and the IC_50_ value was determined to be 108.1 μg / mL (Figure 2-b and Figure 4).

*Wound Healing and Migration Assay*

**Figure 5:**
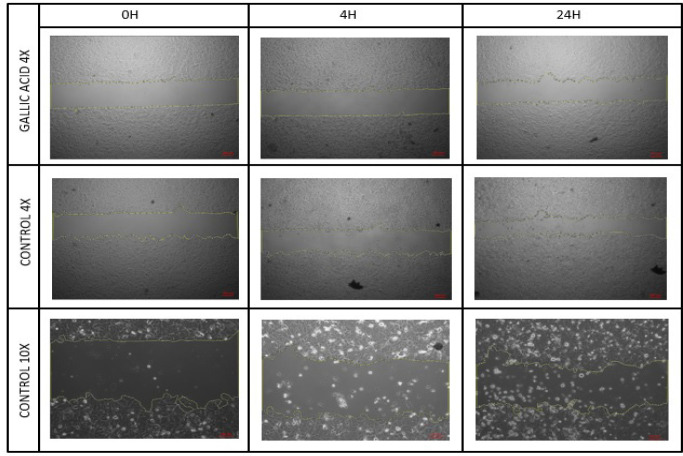
Wound Healing Areas in the Control and Experimental Groups at 0, 4 and 24 Hours

**Figure 6:**
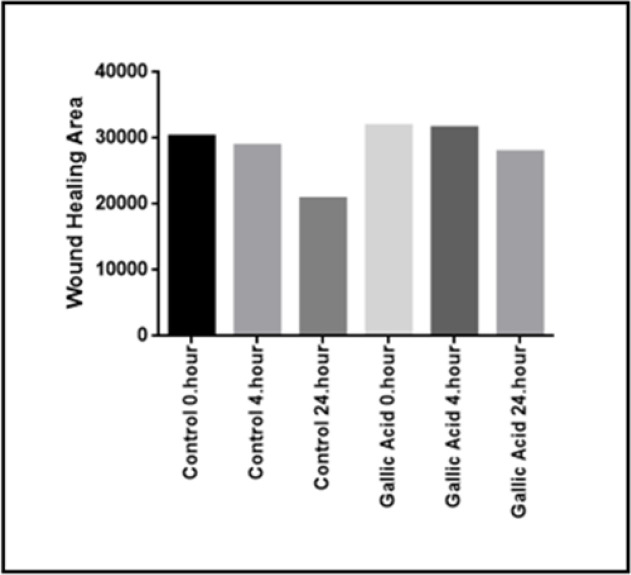
Wound Closure Rates at 0, 4 and 24 Hours

Ishikawa cells were treated with an IC_50_ value obtained in a 2D culture of GA. A cell group which was not exposed to GA was used as the control group and a wound healing experiment was performed. Images were recorded at 0.-4. and 24. Hours (Figure 5). Wound closure rates were determined by field measurement (Figure 6).

*GA causes apoptosis in Ishikawa cells via Caspase 3*

**Figure 7:**
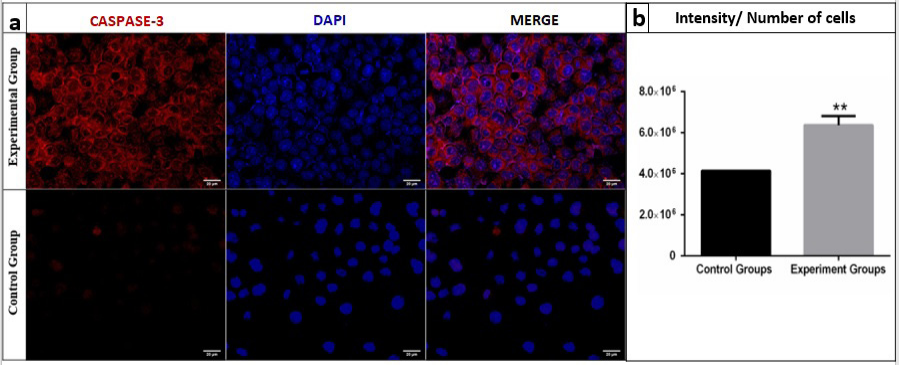
a, Confocal microscopic images of Caspase 3 expressions of the control and experimental groups; b, Caspase 3 expression rates per cell from the control and experimental groups (**: <0.005)

Caspase 3 staining was performed to investigate Caspase 3 activation after GA exposure with a previously determined IC_50_ value on 2D. The images of both groups were recorded by a laser scanning confocal microscope (Figure 7-a) and the Caspase-3 intensities of cells were analysed by an ImageJ program and compared with the control group. Caspase 3 expressions were significantly increased (p=0.0009) in the experimental group (Figure 7-b). 

**Table1:** The Effect of Different Doses of GA on Ishikawa Spheroid Proliferations after 24 hours

	DOSES						
HOURS	5 μg/ml	10 μg/ml	30 μg/ml	50 μg/ml	75 μg/ml	100 μg/ml	CONTROL
0.Hour	471,518 µm	423,881 µm	445,238 µm	417,828 µm	402,506 µm	401,27 µm	489,007 µm
24.Hour	545,374 µm	478,752 µm	507,123 µm	448,459 µm	389,45 µm	375,585 µm	491,997 µm

## Discussion

Although the anticarcinogenic effect of GA has been shown in several cancer cell lines including colorectal(Forester et al., 2014), hepatacellular(Lima et al., 2016) and oral cancers(Guimaraes et al., 2016), its effect on the human endometrium cancer cell line has not been investigated yet. These studies reported varying cytotoxic effects with different doses and for different cell lines. In one of those studies, Maurya and Nandakumar analysed the cytotoxic effect on human lung adenocarcinoma (A549) cells and observed that GA inhibits cell growth up to 200 µM, but that the inhibition effect was not significant at doses above this value(Maurya et al., 2010). 

In the present study we evaluated the effect of different doses of GA on the Ishikawa cell line both in 2D and 3D culture. The cytotoxic effects of 10 μg / ml to 100 μg / ml doses in 2D culture showed a significant increase compared to the negative control group not exposed to GA.

Although 2D culture studies have provided valuable information about the mechanisms underlying malignant growth, in the light of recent data they are not accepted as suitable models to mimic in vivo tumors. Suggested reasons are that solid tumors grow in a three-dimensional spatial plane, in which cells are not homogeneously exposed to nutrients and oxygen, as well as the impact of different physical and chemical conditions. As a result, significant cellular heterogeneity occurs due to the micro-environmental variations found in different regions of solid tumors(Sutherland, 1988). 

In the light of these data, comparative dose-response studies in two and three dimensional cell cultures have gained popularity. Several studies carried out on different cell lines with different compounds have proven the importance of 3D culture by showing a significant difference in cytotoxic doses between two and three dimensional cultures. In one such study, conducted to compare 2D and 3D culture models in the breast cancer cell line (Mcf-7), the cells in 3D models have been shown to be much more resistant to the drugs investigated(Imamura et al., 2015). In another study investigating the effect of the cytotoxic agent Cisplatin in 2D and 3D culture models of Hela cells, the inhibitor concentration in 2D models was significantly lower in 2D models than in 3D models (7.5 µM -60 µM) (Huang et al., 2015). In yet another study with human skin cells (keratinocytes, dermal fibroblasts and endothelial cells), it is reported that the inhibitory concentration was much lower in 2D cultures than in 3D cultures when exposed to silver nitrate, a potentially toxic heavy metal(Sun et al., 2006). These results provided evidence that drug resistance level is higher in 3D culture systems than in 2D models, just as it is in in vivo. 

Spheroids, which are 3D cellular groups, are divided into three different regions according to the cell’s different exposure degrees to the substances by diffusion during culturing. These three regions are the outermost ‘proliferation zone’, in which the diameter growth occurs, the stable ‘living zone’ in the middle, and the ‘necrotic zone’ at the innermost zone(Lin and Chang, 2008). 

In this present study, we analysed the effects of the same doses tested in 2D cultured Ishikawa cells on 3D cultured Ishikawa spheroids. We observed that doses between 5 to 50 μg / ml GA did not trigger any inhibition on the size of spheroids, but instead increased its proliferation, causing an increase in diameter. However, at 75 and 100 μg / ml doses, the cells in the proliferative zone were observed to be significantly detached from the spheroids (Table 1, Figure 3). Because of this, we conducted an MTT analysis for these two doses on spheroids and found a significant increased cytotoxicity in 100 μg / ml doses compared with the negative control group (Figure 2-b). 

These results indicated that the dose in which cytotoxic effect was observed in 2D cultured Ishikawa cells had an opposite effect on 3D cultures, increasing proliferation instead of inhibiting it. The evidence comes from our finding that the IC_50_ value for 2D cell culture was increased by ~13 times in spheroid models from 8.4 μg / ml (Figure 1) to 108.1 μg / ml (Figure 4). The results obtained are compatible with the studies comparing the effects of different molecules between 2D and 3D cultures in different cell lines (Huang et al., 2015). 

We also investigated the effect of GA on the migration profile of 2D cultured Ishikawa cells. For this purpose, an excisional wound model was performed on 2D cultured cells and the cells were then treated with an IC_50_ (8.405 μg / ml) GA dose previously determined in the 2D cell culture. The closure of wound weas visualised and measured at 0, 4 and 24 hours (Figure 5). The wound healing area was observed to be lower than the control group at the end of the 24th hour, although the difference observed was not statistically significant (Figure 6). This may be due to the fact that a 24-hour administration of GA is sufficient to produce a cytotoxic effect but not enough to significantly inhibit the migration of cells.

While analyzing the anticarcinogenic properties of both synthetic and naturally derived compounds, effects are expected to occur through the apoptosis mechanism, or programmed cell death, rather than through necrosis. Caspase 3 is a protein which both the intrinsic and extrinsic pathways of apoptosis use. 

Several studies have reported GA to perform its anticarcinogenic effect via Caspase 3 activation (You, Moon et al. 2010). We applied an IC_50_ GA dose (8.405 μg / ml) to 2D cultured Ishikawa cells, incubated for 24 hours (Figure 1), and observed a significant increase in Caspase 3 expression per cell in the experimental group compared to the control group (Figure 7-a and Figure 7-b). These results support the results of other studies that suggest an increased expression of Caspase 3 after treatment with GA (Ji et al., 2009).

According to our results, we may conclude that GA have anticarcinogenic effects on both 2D and 3D endometrial cell lines in a dose dependent manner. One of the proposed mechanisms that may possibly cause this effect is the triggering of apoptosis via Caspase 3 activation. But apoptosis should be proven further by conducting specific experiments. In addition, the migration of cells should be observed thoroughly with different GA doses and Caspase 3 activation should be performed on 3D culture; these are the main limitations of our study. 

## Author Contribution Statement

Study conception and design: Muhammet Volkan Bulbul, Seda Karabulut, Ilknur Keskin; Data collection: Muhammet Volkan Bulbul, Mervenur Kalender; Analysis and interpretation of results: Muhammet Volkan Bulbul, Seda Karabulut, Mervenur Kalender, Ilknur Keskin; Draft manuscript preparation: Muhammet Volkan Bulbul, Seda Karabulut, Mervenur Kalender, Ilknur Keskin;All authors reviewed the results and approved the final version of the manuscript.
